# Nosocomial Jejunal Mucormycosis - an Unusual Cause of Perforation Peritonitis

**Published:** 2017-07-01

**Authors:** Chandan Kumar, Pragya Jain, Neelam Wadhwa, Preeti Diwaker, Khan Nirupma Panikar

**Affiliations:** 1 *Dept. of Pathology, University College of Medical Sciences, University of Delhi, Delhi, India*; 2 *Dept. of Pathology, Guru Teg Bahadur Hospital, Shahdra, Delhi, India*

**Keywords:** Mucormycosis, Nosocomial Infection Jejunum, Small Bowel

## Abstract

Mucormycosis is a rare but highly invasive opportunistic fungal infection. Gastrointestinal disease although uncommon is highly fatal. We report a case of jejunal mucormycosis in a 24 year old undernourished female with preceding surgical intervention for acute intestinal obstruction of tubercular etiology. On 8^th^ post-operative day, she developed oozing from suture line, prompting exploratory laparotomy, bowel resection, jejunostomy and ileal mucus fistula. Resected bowel showed one perforation and several areas of impending perforations. Characteristic broad, pauci-septate hyaline, empty looking hyphae with infrequent branching were found transmurally and showing angio-invasion. Local intestinal tissue trauma coupled with her sub-normal immune status permitted this unusual nosocomial infection. Histopathological demonstration of the fungus in surgical specimens remains cornerstone of diagnosis of mucormycosis in view of its non-specific symptoms, low isolation rates of mycological culture and lack of other rapid tests.

## Introduction

Mucormycosis is a rare but highly invasive opportunistic infection of ubiquitous fungi of order Mucorales. It is the third most common nosocomial fungal infection following candidiasis and aspergillosis (1). Hyphal forms of the fungus have special affinity for blood vessels and responsible for tissue invasion and disseminat ion. Incidence of mucormycosis is on rise (2, 3). Significant increase in incidence of annual incidence rate of mucormycosis (+7.4%, p<0.001) has been reported by a multicenter study from France (3). This higher frequency has been attributed to surge in high risk patients, especially due to malignancies including recipients of hematopoietic stem cell/solid organ transplant who have surpassed diabetics as the highest at-risk population. Comparative analysis of 2 large studies including patients during 1940-1999 and 1997-2006 respectively, showed that the relative proportion of former risk group increased from 21.5% to 53% while in patients with diabetes reduced from 36% to 16.2% over decades (2, 3). 

 Most common presentation of mucormycosis is rhinocerebral (39-48%) followed by pulmonary (17-24%) and cutaneous (19%). Gastrointestinal (GI) mucormycosis accounts for 7-13% of cases (2,4). Its clinical presentation is non-specific and preoperative diagnosis is difficult. GI mucormycosis has very high mortality as 85-90% die (4). Small intestine is among less commonly involved sites of gut (5). We here report a rare case of nosocomial jejunal mucormycosis leading to perforation peritonitis in a young female with preceding ileal tuberculosis.

## Case Presentation

**Figure 1:**
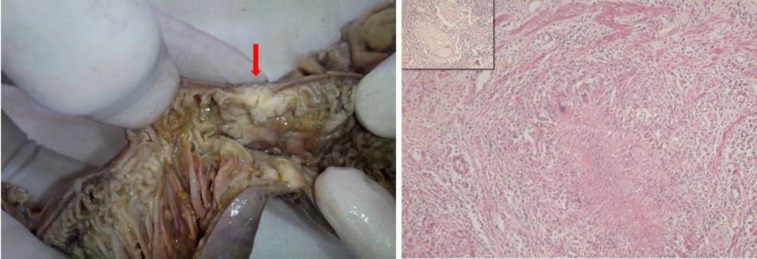
Left: Segment of small bowel from first laparotomy showing a stricture with luminal narrowing (arrow). Right: Epithelioid cell granuloma with central caseous necrosis and Langhans giant cell seen adjacent to muscularis mucosae (HE-100 x). Inset shows granuloma in mesenteric lymph node dissected from the specimen.

A 24-year-old average built, HIV negative underweight (43 kg) woman presented with symptoms of acute bowel obstruction. She complained of fever, multiple episodes of vomiting, abdominal pain, distention and non-passage of stools for three days. Her blood pressure was low (78/44 mm Hg), had tachycardia (114/minute) and tachypnea (24/minute). Clinical examination demonstrated signs of peritonism with distended abdomen, and tenderness and guarding in examination. Erect X-ray abdomen revealed free gas under the diaphragm. Routine hematologic and biochemical parameters were within normal limits except for leukocytosis (16450/mm^3^). Serology for HIV and hepatitis B surface antigen had negative results. The patient underwent exploratory laparotomy for suspected perforation peritonitis. Peritoneal cavity contained approximately two litres of biliopurulent fluid. The affected ileal segment was resected and ileo-ileal anastomosis was performed. The resected small bowel segment (15 cm) showed one stricture with luminal narrowing and three perforations (Fig. 1a). Mesenteric lymph nodes were enlarged and tubercles identified on omentum. Histopathologic examination revealed granulomatous ileitis and mesenteric lymphadenitis with caseous necrosis suggestive of tubercular etiology (Fig. 1b). 

**Figure 2:**
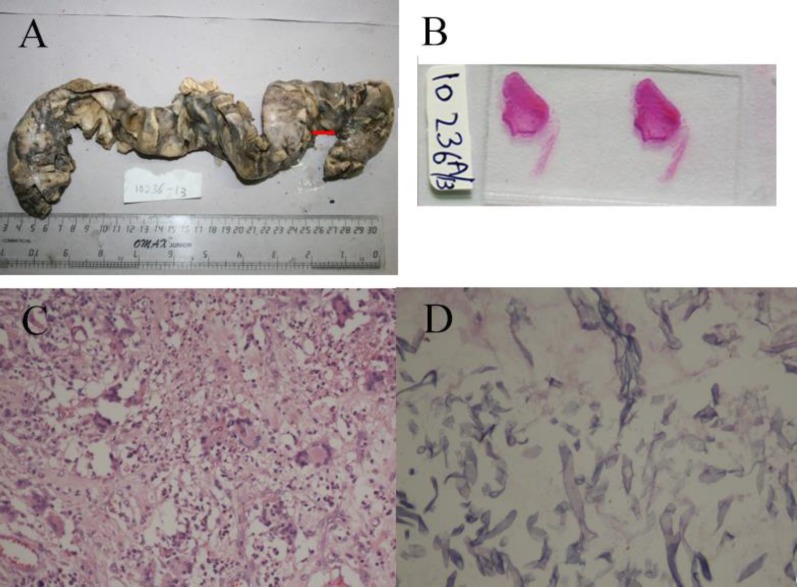
Segment of small bowel from the second laparotomy showing exudate at several places. A perforation (arrow) is also seen near one of the resected ends. B: Marked wall thinning suggestive of impending perforation as seen on naked eye examination of slide. C: Florid giant cell reaction showing several intra-cellular fungi (HE – 200x). D: High power showing typical fungal morphology of aseptate hyphae, section from a thinned out area (PAS – 400x).

**Figure 3:**
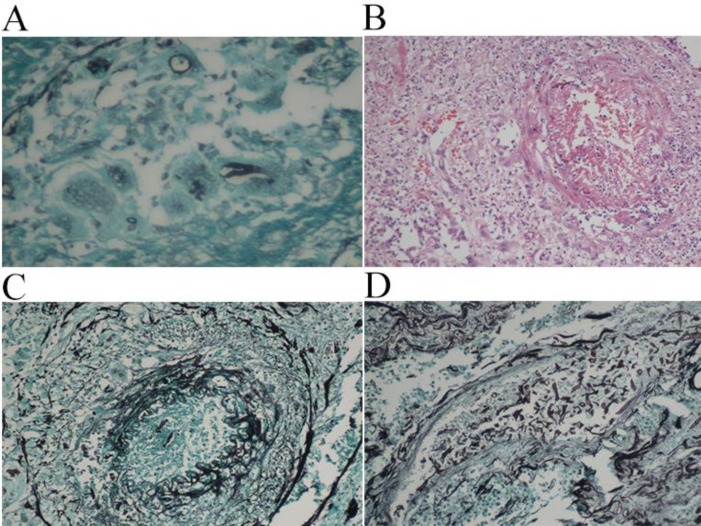
A: Grocott Gomori methanamine silver stain showing broad fungal hyphae engulfed by a giant cell (GMS – 400x). B: Blood vessel showing vasculitis and lumen obliteration by thrombosis (HE – 200x). C: Grocott Gomori methenamine silver stain showing intra-vascular hyphae (GMS – 200x). D: Grocott Gomori methenamine silver stain highlighting the presence of numerous fungal hyphae inside the lumen of a blood vessel (GMS – 200x).

The patient received antitubercular drugs; however, continuous hypotension (80/64 to 92/58 mm Hg) requiring constant ionotropic support and tachycardia (92-104/minute) was evident postoperatively. On eighth postoperative day she developed bilious oozing from the suture line and bilio-purulent fluid from the drain tube prompting clinical diagnosis of postoperative peritonitis. Bedside ultrasound revealed dilated bowel loops and free peritoneal fluid with internal echoes suggestive of peritonitis. The re-look exploratory laparotomy showed anastomotic site leak and one litre biliopurulent fluid in peritoneal cavity. Bowel resection with jejunostomy was performed and ileal mucus fistula made. Resected segment of jejunum was edematous, bile-stained, exudate covered, had one perforation (Fig. 2a) and several areas of wall thinning with impending perforation (Fig. 2b). Microscopic study revealed mucosal ulceration, transmural acute inflammation, focal areas of giant cell reaction and marked serositis (Fig. 2c). Fungal organisms were detected both intra and extra-cellularly (Fig. 2d). The fungal hyphae were broad (6-16μm) with non-parallel sides and focal bulbous dilatations. The hyphae had characteristic empty look with very pale central portions and thin walls. Septation was infrequent, branching non-dichotomous and often at right to wide angles (Fig. 3a). Sections from perforation site showed fungal hyphae infiltrating transmurally. Fungal angio-invasion with vascular thrombosis was also identified at several places (Fig. 3 b-d). Despite thorough sampling, no yeast forms were seen. The hyphae were Periodic Acid Schiff positive and stained dark brown to black with Grocott-Gomori methenamine silver method. These morphological features were consistent with diagnosis of GI mucormycosis. Review of previous sections did not reveal fungal organism. She received Amphotericin B. She was hemodynamically unstable and died on sixth postoperative day of the second surgical intervention.

## Discussion

Mucormycosis is a rare but often fatal opportunistic fungal infection of Mucorales order (1, 2, 4). The term ’zygomycosis’, which was often used interchangeably with mucormycosis has now become redundant. This is due to abolition of phylum Zygomata (in which Mucorales were earlier placed) in the recent molecular phylogenetic classification. Mucorales are now classified under new phylum Glomeromycota (6). Moreover, *Rhizopus* species has been found to be the most frequent culture isolate (34-47%) followed by Mucor (18-19%) (1,2,4,7). 

Mucorales are ubiquitous saprophytic fungi commonly found in soil and decaying matter. Sporangiospores, the infective forms become air-borne easily. Infection is often acquired by inhalation, ingestion or direct inoculation (8). Predisposing factors include typical immune-suppressed conditions such as organ transplantation, hematologic malignancies, neutropenia, diabetes mellitus and burns. Peculiar association with use of iron chelators such as deferoxamine/desferrioxamine for iron or aluminum overload in dialysis patients is also recognized (1,2,4,7,8). Notably 19% to 54.4% of patients with mucormycosis do not have any identifiable predisposing factor (2, 3). Our patient did not have any of the above-mentioned conditions. HIV infection is not a risk factor for mucormycosis; neutrophils rather than lymphocytes are crucial for defence (1). 

Mucormycosis is becoming an important nosocomial fungal infection. Chakrabarty et al. found 9% of their cases to be hospital acquired infections (4). The largest series of mucormycosis attributed to healthcare procedures (169 cases) found a significant association with prior surgical intervention, solid organ transplant and neonatal age (p<0.01 for all). Unlike community acquired infection, skin was the commonest site of localization (57%) followed by GI tract (15%). Contaminated items such as intra-venous catheters, bandages and tongue depressors were identified as possible sources of infection (7). 

GI mucormycosis is uncommon, accounting for 7% to 13% of cases (2,4). Any segment of GI tract may be involved; stomach is the most common site (57.5%) followed by colon (32.2%). Small intestine is less commonly affected (10.3%); jejunum being the least likely site (1.1%) (5). GI involvement is more common in children than adults; in a review of 157 cases of paediatric mucormycosis, Zaoutis et al. found GIT disease (21%) to out-number rhinocerebral involvement (18%) (9). Moreover, Chakrabarty et al. found 70% of their GI mucormycosis cases in children (4). Neonatal GI mucormycosis is a unique category; pre-term neonates are at risk, colon is typically involved and clinical presentation resembles necrotizing enterocolitis (10). 

Risk factors for GI mucormycosis may not include typical immune-compromised status such as malnutrition or alcoholism. Preceding mucosal trauma such as ulceration due to typhoid, amoebic colitis and post-surgical states have been documented (8). This suggests either deposition of air-borne infective spongiophores or ingestion and their germination/sporulation due to poor local immune defence. Mini-outbreaks of gastric mucormycosis in hospitals have been found to be associated with the use of contaminated wooden tongue depressors (11). Our patient had acquired immunosuppression owing to malnutrition with negative results for HIV serology. She also had mucosal compromise consequent to her earlier surgery. Till date, mucormycosis of the jejunum has been described in two cases (12,13). However, to the best of our knowledge, this was the first reported case of nosocomial jejunal mucormycosis following GI tuberculosis. 

Clinical presentation of GI mucormycosis is non-specific. Abdominal pain, hematemesis and bleeding per rectum are commonly described. Perforation and peritonitis are secondary to its hallmark of hyphal vascular invasion leading to endarteritis, thrombosis, tissue ischemia and infarction (7). Our case had anastomotic site leak, mucosal ulceration and several impending perforation sites in the jejunum, which correlated well with angio-invasion seen on microscopy. 

Due to its rarity, mucormycosis is seldom suspected preoperatively and mycologic cultures often unavailable. Although useful for species identification and drug susceptibility tests, culture cannot differentiate invasive infection and innocuous contamination, unless isolated repeatedly from sterile body sites (7). Culture isolation rates vary between 50% and 71% (2). Hence, histopathologic diagnosis remains cornerstone of invasive mucormycosis. Shorter turn-around time, its ability to identify unsuspected and culture negative cases are other advantages (7,10). The morphology of invasive mucormycosis is quite characteristic (7). The fungal hyphae are typically hyaline, broad, pauci-septate and have non-parallel thin walls with central pallor giving them an empty look. Branching is infrequent and at right to obtuse angle. These features help in excluding aspergillosis. The hyphae of latter are septate, uniformly thinner (2-3m), with parallel edges showing regular dichotomous branching at 45^0^. Rare post-therapy cases of aspergillosis may show widening of hyphae but wide angle branching is not evident. Candida species show thin pseudo-hyphae with constriction at septation site. Yeast forms are also easily seen. In tissues, mucormycosis does not exhibit dimorphic morphology. Larger hyphae size and other morphologic features help in excluding candidiasis. Special stains such as Periodic acid Schiff (PAS) with diastase and silver stains are useful for highlighting organism morphology and demonstration of vascular invasion. 

GI mucormycosis is often lethal and 85% to 90% of patients die (2, 3). Bowel perforation and malnutrition are strongly associated with death (2). Major factors in mitigating mortality in mucormycosis are early diagnosis, correction of underlying predisposing factors, surgical debridement and prompt anti-fungal therapy (1,2). Despite resection of affected bowel segment and timely initiation of anti-fungal treatment, our patient had a fulminant course. Possibility of disseminated mucormycosis cannot be excluded in our case as a reason for sustained hypotension which remained non-responsive to ionotropic support. 

## Conclusion

We here shared our experience of an uncommon case of nosocomial jejunal mucormycosis presenting as anastomotic site leak and perforation peritonitis in a patient with malnutrition and antecedent tubercular ileitis. Mucormycosis should be considered as a possible etiology of anastomotic site leak in patients with sub-optimum immune status like those with malnourishment. Endoscopy (although not performed in our case) is a less invasive technique for early diagnosis. Prompt surgical debridement and anti-fungal treatment lower mortality rate. Failure to show expected recovery or sudden clinical deterioration, protracted and unexplained hypotension, as happened in our case are pointers to unresolved issues.
